# Diagnostic Value of CT Arthrography for Evaluation of Osteochondral Lesions at the Ankle

**DOI:** 10.1155/2016/3594253

**Published:** 2016-11-07

**Authors:** Jan S. Kirschke, Sepp Braun, Thomas Baum, Christian Holwein, Christoph Schaeffeler, Andreas B. Imhoff, Ernst J. Rummeny, Klaus Woertler, Pia M. Jungmann

**Affiliations:** ^1^Department of Neuroradiology, Klinikum Rechts der Isar, Technische Universität München, Ismaninger Strasse 22, 81675 Munich, Germany; ^2^Department of Orthopaedic Sports Medicine, Klinikum Rechts der Isar, Technische Universität München, Ismaninger Strasse 22, 81675 Munich, Germany; ^3^Department of Radiology, Klinikum Rechts der Isar, Technische Universität München, Ismaninger Strasse 22, 81675 Munich, Germany; ^4^Department of Trauma and Orthopaedic Surgery, BG Unfallklinik Murnau, Professor-Küntscher-Strasse 8, 82418 Murnau, Germany; ^5^Musculoskeletal Imaging, Kantonsspital Graubuenden, Loestrasse 170, 7000 Chur, Switzerland

## Abstract

*Background*. To retrospectively determine the diagnostic value of computed tomography arthrography (CTA) of the ankle in the evaluation of (osteo)chondral lesions in comparison to conventional magnetic resonance imaging (MRI) and intraoperative findings.* Methods*. A total of *N* = 79 patients had CTAs and MRI of the ankle; in 17/79 cases surgical reports with statements on cartilage integrity were available. Cartilage lesions and bony defects at talus and tibia were scored according to defect depth and size by two radiologists. Statistical analysis included sensitivity analyses and Cohen's kappa calculations.* Results*. On CTA, 41/79 and 31/79 patients had full thickness cartilage defects at the talus and at the tibia, respectively. MRI was able to detect 54% of these defects. For the detection of full thickness cartilage lesions, interobserver agreement was substantial (0.72 ± 0.05) for CTA and moderate (0.55 ± 0.07) for MRI. In surgical reports, 88–92% and 46–62% of full thickness defects detected by CTA and MRI were described. CTA findings changed the further clinical management in 15.4% of cases.* Conclusions*. As compared to conventional MRI, CTA improves detection and visualization of cartilage defects at the ankle and is a relevant tool for treatment decisions in unclear cases.

## 1. Introduction

Osteochondral lesions at the ankle frequently occur after traumatic injuries [1–3]. They represent the most important joint-related risk factors for osteoarthritis at the ankle [1, 2, 4]. Cartilage repair procedures have increasingly been applied at the ankle joint, aiming to decelerate progression to early osteoarthritis.

In order to visualize osteochondral lesions at the ankle and to decide on the treatment strategy and ideal surgical approach, cross-sectional imaging is required. Usually conventional magnetic resonance imaging (MRI) is considered as the modality of choice [5]. However, in comparison to the knee joint, articular cartilage at the ankle joint is very thin (0.4–2.1 mm) [6–10]. Therefore assessment of morphological cartilage defects is challenging [10]. Low sensitivities for detection of osteochondral lesions at the ankle on MRI were reported, which varied between 50% at 1.5 T and 75% at 3.0 T [8, 11].

CT arthrography (CTA) is a well-established cross-sectional imaging technique for detection of osteochondral lesions in different joints. Also postoperatively, it allows more specific evaluation of equivocal or difficult lesions [5, 12–16]. While, for other joints, high resolution MRI has replaced CTA, for the elbow and the ankle joint, CTA remains clinically relevant [6, 13, 17, 18]. In 2003 Schmid et al. found that CTA was more reliable than MR arthrography (MRA) in the ability to detect osteochondral lesions at the ankle [6]. El-Khoury et al. performed a cadaver study and concluded that cartilage thickness measurements at the ankle were more accurate on CTA than on conventional MRI [19]. Despite its clinical relevance over decades, there is no clinical study that compares ankle CTA performance in comparison to conventional MRI with respect to evaluation of osteochondral defects.

Therefore, the purpose of this study was to determine the diagnostic value and the reliability of CTA at the ankle in the evaluation of osteochondral defects in comparison to conventional MRI. We hypothesized that assessment of osteochondral defects at the ankle is more reliable on CTA than on MRI and that CTA of the ankle has a high diagnostic relevance in order to determine the surgical approach.

## 2. Materials and Methods

### 2.1. Subjects

The work was conducted in accordance with the Declaration of Helsinki. The study was approved by our institutional review board. The requirement for informed consent was waived. The data of patients referred for multidetector CT of the ankle at our hospital between July 2004 and July 2015 were evaluated retrospectively. Inclusion criteria for this study were CTA of the ankle performed at our institution and an available MRI examination of the same ankle performed within 12 months prior to or 3 months after the CTA (*n* = 3 patients received CTA > 6 months after MRI). Subjects were excluded, if surgery was performed between acquisition of MRI and CTA. Further, subjects with CT scans of the ankle without arthrography, as well as subjects without MR examinations, were excluded. Finally the study cohort consisted of *N* = 79 subjects.

### 2.2. Contrast Injection

Intra-articular injection of contrast media was performed under fluoroscopic guidance by means of a medial approach [20]. Intra-articular positioning of the needle (20- to 22-gauge needle; Terumo, Belgium) was confirmed by injection of a small amount of iodinated contrast media. For CTA, 6–8 mL of a solution consisting of iodinated contrast media (Ultravist 300; Schering, Berlin, Germany) and saline (2 : 1) was injected. CT imaging was performed within 15 minutes after contrast agent injection.

### 2.3. CT Imaging

CT images were acquired by using either a clinical whole-body 256-row CT scanner (Philips Brilliance iCT, Philips Medical System DMC GmbH, Hamburg, Germany) or a clinical 128-row Siemens SOMATOM Definition AS (Siemens Healthcare, Erlangen, Germany). Scan parameters were as follows: tube voltage, 120 kVp; tube load, 250 mAs; interpolated voxel size, 300 × 300 × 600 *μ*m^3^; feed/rotation, 4.8 mm; single collimation width, 0.3; and effective pitch, 1.0. For reconstruction a 360° interpolation algorithm and a high resolution kernel (U70v very sharp) were used. Transverse, coronal, and sagittal reformations were obtained with a reconstruction increment of 2.0 mm, a window center of 500 HU, and a window width of 2500 HU.

### 2.4. MR Imaging

MR imaging protocols varied, since some of the patients were referred to the sports orthopedics department with existing MR examinations. MR imaging was predominantly performed on 1.5 T systems (Magnetom Avanto, Siemens AG, Erlangen, Germany) or 3 T systems (e.g., Siemens Verio, Global Siemens Healthcare Headquarters, Siemens AG, Erlangen, Germany). Siemens Head/Neck 4-channel coils were used (Global Siemens Healthcare Headquarters, Siemens AG, Erlangen, Germany). Exemplary MR imaging parameters at 3 T are given in Table 1.

### 2.5. Image Analysis

MR images were transferred on Picture Archiving Communication System (PACS) workstations (Easy Vision, Philips, Best, Netherlands) and were evaluated semiquantitatively by two musculoskeletal radiologists independently (Jan S. Kirschke and Pia M. Jungmann). Both observers evaluated all images in a randomized order; MR evaluation was performed before CTA evaluation. For cartilage evaluation and evaluation of the subchondral bone on MRI, primarily IM-w sequences and T1-w sequences were considered.

### 2.6. Semiquantitative Assessment

Osteochondral lesions at the tibia and talus were scored on CTA and MRI including the parameters cartilage defect depth, cartilage defect size, bony defect depth, and bony defect size. Cartilage defect depth was scored as follows: (i) no defect, (ii) partial thickness defect, and (iii) full thickness defect. Cartilage defect size was determined by measuring the largest cartilage defect diameter: one measurement was performed for full thickness parts of the lesion and a second measurement for the entire cartilage lesion. Defect size was scored as follows: no defect, ≤2 mm (fissure), >2 mm and ≤5 mm (small), >5 mm and ≤10 mm (medium), >10 mm and ≤15 mm (large), and >15 mm (extensive). With respect to subchondral bone, size of laminar defects was determined using the same measures as for cartilage lesions. Depth of bony defects was graded as follows: no bony defect; bony defect depth ≤3 mm (superficial); and bony defect depth >3 mm (deep). On MRI, bony defects were assessed on T1-weighted images. ICRS scores [21] and WORMS cartilage scores [22, 23] were assessed additionally. Scores were performed as described previously, except that signal alterations were not scored.

### 2.7. Clinical Relevance

All images were assessed by one specialized orthopedic surgeon (Sepp Braun) in consensus with one musculoskeletal radiologist (Pia M. Jungmann). Changes in the therapeutical or surgical approach resulting from the additional information gained from CTA were noted as follows: (i) no additional information, (ii) influence of CTA findings on therapeutical strategy or surgical approach, and (iii) no influence on the therapeutical strategy but important information and confirmation of the chosen strategy.

### 2.8. Surgical Reports

By means of digital medical records, information on patient demographics, indication for CTA, and information on previous surgeries were retrieved. Surgical reports were analyzed retrospectively regarding description of cartilage integrity. Presence of cartilage defects was noted. Due to missing information on osseous involvement this parameter was not included in the analyses.

### 2.9. Statistical Analysis

Statistical processing was performed with SPSS version 17.0 (SPSS Institute, Chicago, IL, USA) (Pia M. Jungmann and Thomas Baum). Frequencies of subscores in our analyzed cohort were calculated. Means ± SD and means ± SEM were calculated as indicated. Interobserver reliability was determined using Cohen's kappa statistics and was classified as suggested by Landis and Koch [24]. Using CTA as gold standard, sensitivity and specificity were determined for MRI. Using surgical reports as gold standard, sensitivity and specificity were determined for both CTA and MRI. All tests were performed based on a 0.05 level of significance.

## 3. Results

### 3.1. Demographics

Between July 2004 and July 2015, *n* = 1649 CT scans of the ankle were performed, of which *n* = 164 were CT arthrographies; *n* = 79 of the subjects had additional MRI scans of the ankle and were included in the study. The mean age was 33.8 ± 13.9 years (*n* = 43 male, *n* = 36 female). In *n* = 34 cases the right ankle was examined; in *n* = 43 cases the left ankle was examined. In 68/79 cases, MRI was performed before CTA. The mean time interval between CTA and MRI was 2.8 ± 3.0 months. Consecutive surgery with description of cartilage integrity in the surgical report was performed in 17/79 subjects. The mean interval between CTA and surgery was 2.7 ± 3.2 months. The mean interval between MRI and surgery was 3.5 ± 3.1 months.

### 3.2. Indications and Surgeries

**Figure 1:**
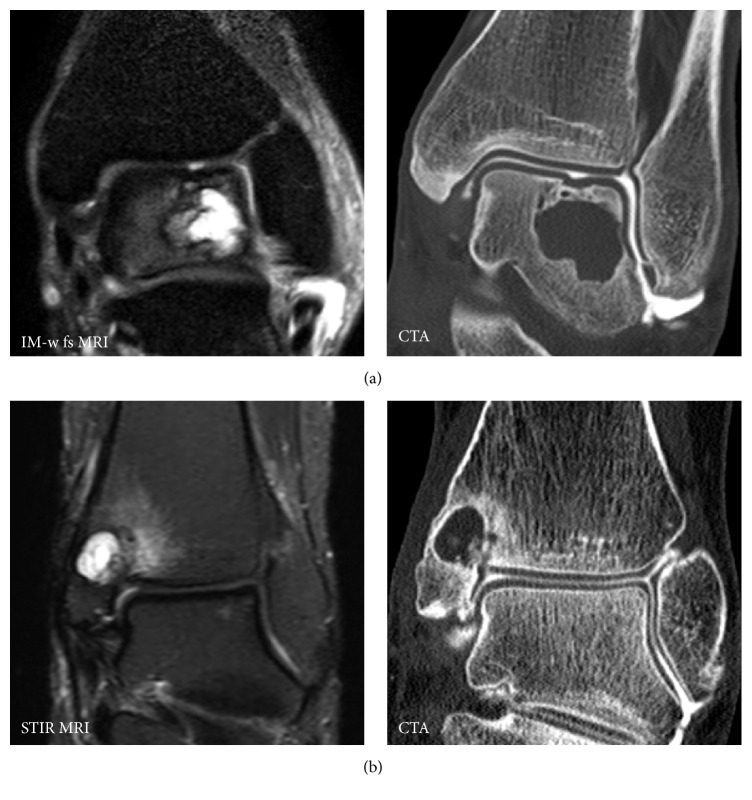
CTA for detection of full thickness cartilage lesions at the ankle in the presence of subchondral cysts. On MRI presence of full thickness cartilage defects remain unclear. In case (a) CTA demonstrates incongruence of the cartilage surface but no full thickness defect. In case (b) CTA demonstrated a fissural full thickness cartilage defect, allowing communication between intra-articular synovial fluid and subchondral cyst.

**Figure 2:**
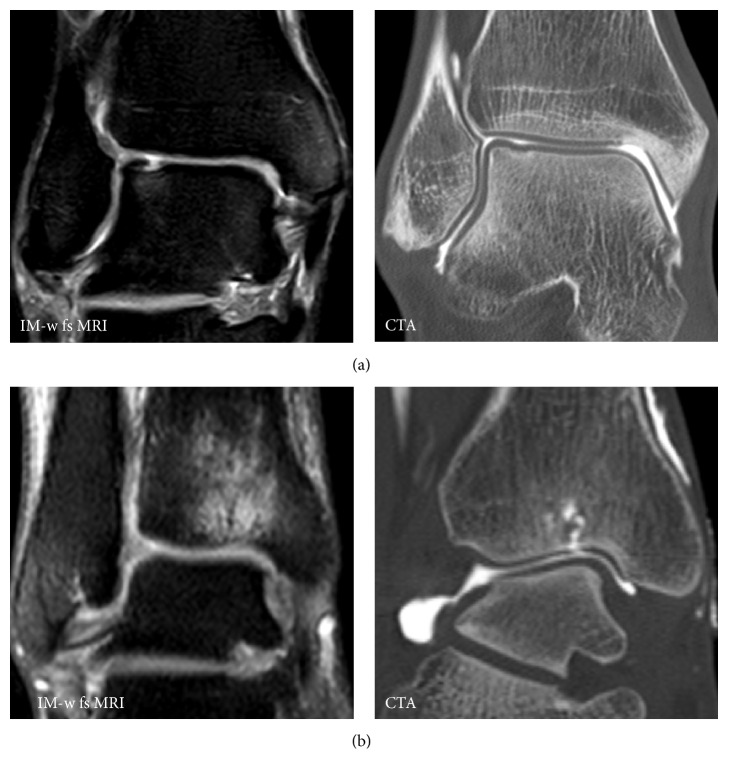
Divergent results for presence of full thickness cartilage defects on MRI and CTA. In case (a) a full thickness cartilage defect at the talus was suggested on MRI; CTA revealed depression of the cartilage surface but no full thickness defect. In case (b) no cartilage defect was suggested on MRI but due to massive bone marrow edema at the tibia CTA was performed and revealed a fissural full thickness defect at the tibia.

**Figure 3:**
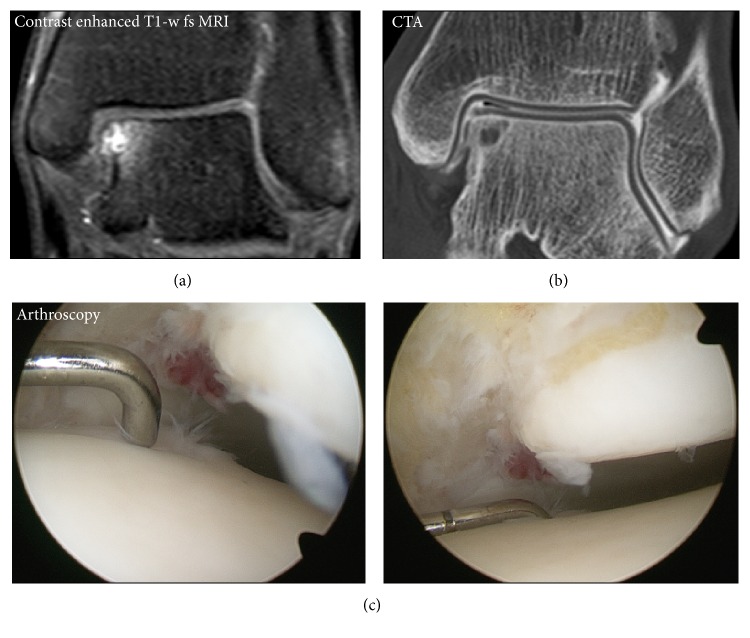
Considering intraoperative findings as standard of reference. CTA showed improved sensitivity compared to MRI. Exemplarily, in this subject with an osteochondral defect at the medial talus only BMEL but no cartilage defect was depicted on MRI (a). The full thickness cartilage defect revealed by CTA (b) was confirmed with a probe intraoperatively (c).

**Figure 4:**
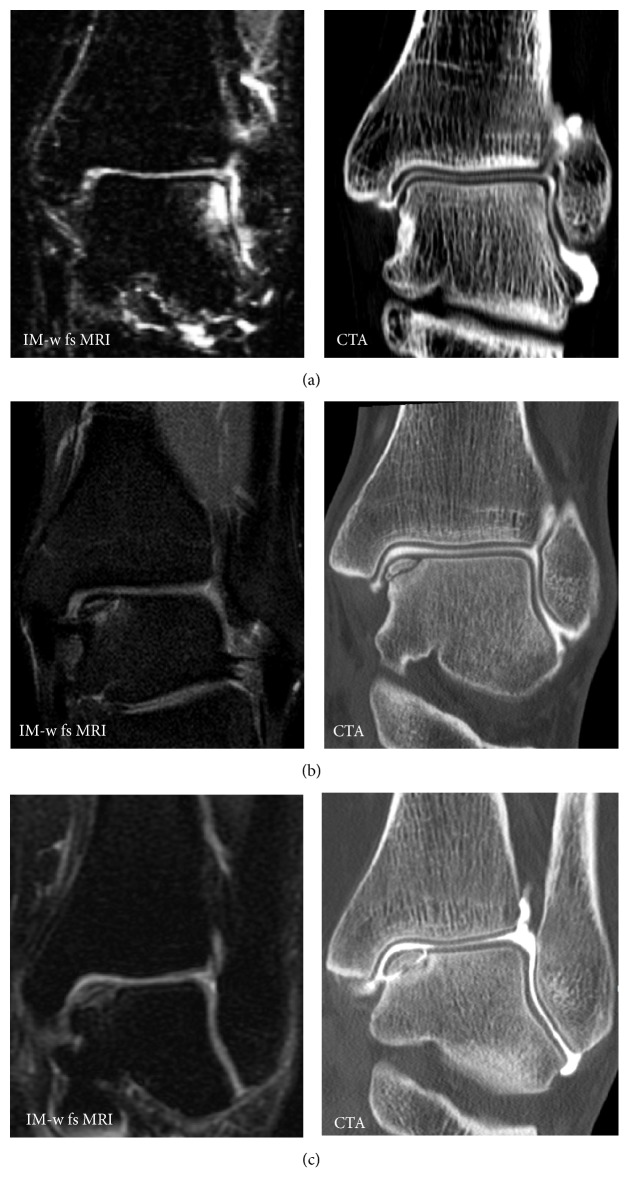
Different presentations of osteochondral defects. (a) Patient with suspicion of an osteochondral defect on MRI but no defect on CTA. (b) Patient with an osteochondral defect without loosening (no subsequent surgery). (c) Patient with similar findings on MRI as in (b); however on CTA contrast enhanced fluid surrounds the osteochondral fragment indicating instability and the patient had to undergo subsequent surgery.

In all cases, the CTA was performed in order to detect or visualize chondral or osteochondral defects. Specific indications for CTA, previous surgeries, and surgeries after CTA are given in Table 2 and Figures 1, 2, 3, and 4. *N* = 9 patients had metal implants; *n* = 8 additional patients showed susceptibility artifacts on MRI. Of note, in two cases contrast was diminished due to a long gap between contrast application and CT scan; however, we did not exclude these patients not to cause any bias. To the best of our knowledge, none of our patients experienced any adverse effects from the intervention.

### 3.3. Frequencies of Lesions

Frequencies of lesions in the assessed cohort are given in Table 3. On CTA 51/79 patients had cartilage defects at the talus, of which 41/51 were graded as full thickness cartilage defects. At the tibia, 38/79 patients had cartilage defects, of which 31/38 were full thickness cartilage defects. In *n* = 10 cases (12.7%) no morphological defect was seen on CTA. In subjects with BMEL or cysts as indication for CTA, two-thirds (14/22 cases) showed full thickness cartilage defects on CTA (cysts, 10/15; BMEL, 4/7; Figures 1, 2, and 3). On CTA 41/79 (38%) subjects had lesions of the subchondral bone at the talus and 30/79 (52%) had lesions of the subchondral bone at the tibia. On MRI 55/79 patients had cartilage defects at the talus (tibia: 41/79), of which 36/55 were graded as full thickness defects (tibia: 20/41). In *n* = 15 cases (19.0%) no cartilage defect was suspected on MRI; in 11/15 of those cases defects were detected on CTA in the following. Mean ICRS scores were 2.1 ± 1.9 on CTA and 2.1 ± 1.8 on MRI. Mean WORMS scores were 1.6 ± 1.5 on CTA and 1.6 ± 1.5 on MRI. Cartilage defects at the talus and at the tibia were found in 64.7% and 29.4% of surgical procedures, respectively.

### 3.4. Interobserver Reliability

Kappa values for interobserver reliability are presented in Table 4. Agreement was substantial for presence of full thickness cartilage defects on CTA (mean ± SEM, 0.72 ± 0.05) and moderate for presence of full thickness cartilage defects on MRI (0.55 ± 0.06). For presence of bony lesions substantial agreement was found for CTA (0.70 ± 0.06) and MRI (0.78 ± 0.04). Interobserver agreement for semiquantitative ICRS and WORMS scores was substantial for CTA (0.71 ± 0.05 and 0.61 ± 0.05) and moderate to substantial on MRI (0.62 ± 0.04 and 0.54 ± 0.04).

### 3.5. Diagnostic Sensitivity

Considering CTA as standard of reference, MRI was able to visualize 83.1% of cartilage defects (observer 2, 67.0%; specificity, 68.1% and 85.2%); 54.2% of full thickness defects were depicted (observer 2, 47.6%; specificity, 80.2% and 86.5%). MRI was able to visualize 63.6% of defects of the subchondral bone. Considering surgical reports as standard of reference, sensitivity for detection of cartilage lesions was better for CTA than for MRI (Figure 3). CTA was able to visualize 87.5% of cartilage defects (observer 2, 92.3%; specificity, 55.6% and 38.9%); 84.6% of full thickness defects were depicted (observer 2, 92.3%; specificity, 74.1% and 52.4%). MRI was able to visualize 81.2% (observer 2, 75.0%; specificity, 55.6% and 66.7%) of cartilage defects; 46.2% of full thickness defects were depicted (observer 2, 61.5%; specificity, 76.2% and 85.7%).

### 3.6. Clinical Relevance

In 12/79 cases (15.4%) CTA findings changed the further clinical management of the patient. In *n* = 2 cases, MRI findings did not indicate surgery but CTA findings did; finally these patients had subsequent surgery. In *n* = 3 cases MRI findings indicated surgery, but CTA did not; finally these patients had no surgery. In *n* = 1 case CTA findings changed the surgical approach. In *n* = 6 cases, CTA findings confirmed the therapeutic decision based on MRI and did avoid diagnostic arthroscopy. In most cases visualization of the cartilage surface was of additional value for orthopedic surgeons.

## 4. Discussion

The present study demonstrated the important diagnostic value of CTA with respect to osteochondral lesions at the ankle joint, particularly in case of full thickness cartilage lesions. Only about half of full thickness cartilage lesions detected on CTA were depicted on MRI. Interobserver agreement for detection of cartilage lesions and for semiquantitative ICRS and WORMS scores was superior for CTA as compared to MRI. Sensitivity for detection of intraoperatively confirmed cartilage lesions was better for CTA than for MRI. CTA findings were considered beneficial for treatment decisions. These results suggest that in indicated cases CTA of the ankle remains an extremely helpful cross-sectional imaging tool for detection, visualization, and scoring of chondral and osteochondral lesions at the ankle joint.

While there are many cohort studies on cross-sectional imaging of early knee osteoarthritis, there is a lack of imaging studies on the ankle joint. Detection of osteochondral defects at the ankle is clinically of particular importance, since these predispose for osteoarthritis [1, 2, 4]. MRI is routinely used to detect chondral and osteochondral defects [25]. Despite some limitations, fairly good correlations of MRI grading of osteochondral lesions at the talus with arthroscopic findings were reported [26, 27]. However, both studies only assessed high-grade osteochondral lesions and only relate to the differentiation between loose and nonloose fragments. Cha et al. only found a sensitivity of 46% for detection of osteochondral lesions at talus on MRI [11]. The authors stated that although intra-articular lesions are usually diagnosed with MRI, its sensitivity and interobserver reliability are low and they recommended additional arthroscopic examination.

Besides MRI, CTA has been used for detection of osteochondral lesions in many joints [12, 17, 18, 28–30]. CTA of the ankle joint is still clinically relevant [16] and the diagnostic value may be underrepresented in the literature. Using the terms “CT arthrography” and “ankle” in a PubMed search, only 49 studies were found. Kraniotis et al. described that CTA detects radiographically silent osteochondral lesions in patients with fractures of the ankle joint but did not compare CTA to other imaging techniques [16]. In the study by Chemouni et al. CT arthrography showed an accuracy of 88% [31]. For detection of retropatellar osteochondral lesions, CTA performed superior compared with conventional MRI in several studies [1, 28]. For detection of osteochondral lesions at the ankle, Schmid et al. reported that CTA was superior to MRA [6]. They reported an accuracy of 90–92% for detection of osteochondral defects at the talus on CTA (76–88% for MRA) with a kappa value for interobserver agreement of 0.69 (0.47 for MR arthrography). In contrast to Schmid et al. we did not use a panel as standard of reference. The authors also doubted its value, since the original readers were already well qualified and the panelists did not perform much better. Instead, in the present study interobserver agreement and surgical reports (of a subcohort of 17/79 cases that received surgery) were used as an established standard of reference, which better represents imaging performance. In an additional analysis CTA was used as a standard of reference and the accuracy of MRI with respect to detection of cartilage defects was evaluated. Despite the methodological limitation of choosing this imaging modality itself as the reference standard, it demonstrates the ability of CTA to depict particularly more full thickness cartilage defects than MRI.

Since BMEL may only be depicted properly on MRI but frequently correlate with patients symptoms, this underlines the value of MRI in this context. In our study, BMEL frequently indicated fissural cartilage defects on CTA (Figure 2). It is known that BMEL are associated with cartilage defects and that the adjacent articular cartilage should be evaluated carefully [6, 27, 32]. Agreement for defect size was moderate for both CTA and MRI. Probably, this is due to the fact that it remains unclear whether unsharp borders of cartilage defects should be included in cartilage defect size measurements. Nevertheless, we found an improved performance for CTA compared with conventional MRI regarding detection of cartilage defects. One explanation might be the relevance of chemical shift artifacts in the ankle joint, which obscure parts of the thin cartilage layer [6, 10]. However, in recent years, improvements of cartilage MR imaging were described. In particular, 3 T MRI is superior to 1.5 T MRI [8, 33, 34]. Improved visualization of the cartilage surface in high resolution MRI of the ankle was achieved by application of a traction device [10]. Comparing CTA with high resolution MR imaging would supposedly decrease the difference between the two imaging techniques.

Cross-sectional imaging at the ankle is required for osteochondral defects visualization and staging not only prior to surgery but also after surgery [6, 35]. In our study, *n* = 33 patients had previous surgery at the ankle (before CTA). A subset of *n* = 9 patients had metal implants. With presence of metal implants, despite 1.5 T MR imaging and conventional and advanced metal artifact reducing sequences, MRI remains challenging [36]. In several studies, it has been shown that IM-w fs sequences perform best with respect to cartilage evaluation [8, 33]. However, spectral fat saturation is very sensitive to magnetic field inhomogeneities and does not work properly in case of presence of metal implants [37]. Therefore in case of metal implants CTA of the ankle remains extremely relevant.

Another important indication for CTA was presence of subchondral cysts. On MRI it was hard or impossible to detect fissural defects that allow communication between intra-articular synovial fluid and cyst. In 66% of cases with subchondral cysts, fissures were detected on CTA (Figure 1). Previously, this finding did influence the choice between antegrade or retrograde surgical approach [6, 35]. Due to recent improvements in cartilage repair procedures, currently the lesions are addressed with an antegrade approach disregarding presence of a transchondral fissure. This explains why, in only 15.4% of cases, the therapeutical management approach changed due to CTA findings. Nevertheless, proper visualization of the osteochondral defect was extremely helpful for orthopedic surgeons.

The major limitation of the present study is the retrospective design. Due to the retrospective design, there was no standardized reporting of osteochondral defects in the surgical report. Supposedly, some minor cartilage lesions, in particular at the opposing compartment, may not have been described in surgical reports since no surgical treatment was required. This may account for the relatively low specificity of both CTA and MRI, using surgical reports as standard of reference. The involvement of the subchondral bone was reported very inconsistently in the surgical reports and was therefore not included in the present study. Only indicated CTAs were performed and included in this study and indications varied. In most cases, CTA was indicated because MRI findings were inconclusive. This may have impacted the results. However, we think that this is an appropriate clinical practice. In case of indicated cases, important additional diagnostic information may be gained via CTA with respect to osteochondral lesions. In *n* = 10 cases no cartilage defect was detected on CTA and CTA did therefore avoid diagnostic arthroscopy. It needs to be stated that CTA may not replace MRI, in particular with respect to detection of pathologies of other joint structures. A second limitation is the varying MRI quality and the differences in MR imaging protocols due to different referring centers. However, the assessed cohort represents a realistic sample of MR images that orthopedic surgeons are routinely confronted with and on which diagnosis, treatment decisions, and surgery planning were based. Further, specificity values given in this study need to be interpreted with caution due to few negative surgeries.

## 5. Conclusions

In summary, this study underlined the important diagnostic value of CTA at the ankle. CTA showed improved sensitivity and reliability regarding detection of osteochondral lesions at the ankle compared with conventional MRI. CTA findings may influence treatment strategies and surgical decisions in many cases. In conclusion, in the appropriate clinical context, CTA is particularly helpful in patients with suspicion of osteochondral lesions at the ankle.

## Figures and Tables

**Table 1:** Exemplary 3 T MR imaging parameters.

Sequence	2D IM-w TSE	2D T2-w TSE	2D IM-w TSE	2D T1-w TSE
Additional features	FS, BLADE		FS, BLADE	DRIVE
Plane	Sagittal	Transverse	Coronal	Coronal
Echo time (TE; ms)	46	77	47	13
Repetition time (TR; ms)	4500	5360	4500	1000
Field of view (FOV; mm)	140	120	140	140
Slice thickness (mm)	3	3	3	3
In-plane resolution (mm^2^)	0.44 × 0.44	0.38 × 0.38	0.36 × 0.36	0.36 × 0.36
Flip angle (°)	90°	90°	90°	90°
Number of slices	22	25	24	22
Receiver bandwidth (Hz/ pixel)	182	147	181	171
Echo train length	9	15	9	3
Phase encoding direction	AP	RL	RL	RL
Distance factor (%)	10	20	10	10
Acquisition time (min)	5 : 08	3 : 57	5 : 48	4 : 04

FOV: field of view; w: weighted; TSE: turbo spin echo; IM: intermediate; fs: fat saturated; BLADE: motion correction with radial blades; DRIVE pulse: driven equilibrium pulse.

**Table 2:** Detailed specifications of the indications for CTA and surgeries.

A: indication	Number of cases (*n* = 79)
OCL → fragment instability? (Figure 4)	*n* = 21
OCL → extend?	*n* = 16
Subchondral cyst or ganglia → fissural defect? (Figure 1)	*n* = 13
Subchondral BMEL → fissural/cartilage defect? (Figure 2)	*n* = 7
After cartilage repair surgery	*n* = 13
Bone necrosis/infarct → cartilage involvement?	*n* = 3
Tumor^a^	*n* = 2
No findings on MRI but persistent complaints → cartilage defect?	*n* = 4

B: previous surgery	Number of cases (*n* = 33)

Osteochondral transplantation	*n* = 9
Autologous chondrocyte implantation	*n* = 2
Biomatrix implantation	*n* = 2
Retrograde drilling	*n* = 5
Curettage of ganglion/cyst and spongiosa graft	*n* = 4
Surgical treatment of ankle fractures	*n* = 7
Tumor surgery (giant cell tumor)	*n* = 1
Arthroscopy (debridement, shaving)	*n* = 3

C: surgery after CTA	Number of cases (*n* = 17)

Osteochondral transplantation	*n* = 2
Microfracturing/antegrade drilling	*n* = 3
Spongiosa graft	*n* = 5
Only chondral and osseous debridement	*n* = 4
Metal implant removal	*n* = 2
Syndesmosis reconstruction	*n* = 1

A: indications including clinical queries (→); B: previous surgeries; C: surgeries after CTA.

^a^Tumors were one chondroblastoma at the talus and one giant cell tumor at the tibia.

OCL: osteochondral lesion; BMEL: bone marrow edema-like lesion.

**Table 3:** Frequencies of osteochondral lesions on CTA and MRI.

	CTA (*n* = 79)		MRI (*n* = 79)	
	Talus	Tibia	Talus	Tibia
	*n* (% of “*total*”)	*n* (% of “*total*”)	*n* (% of “*total*”)	*n* (% of “*total*”)
*Full thickness cartilage defect*				
Total	*n* = 41	*n* = 31	*n* = 36	*n* = 20
Fissure	*n* = 16 (39%)	*n* = 16 (52%)	*n* = 6 (17%)	*n* = 4 (20%)
Small	*n* = 9 (22%)	*n* = 6 (19%)	*n* = 11 (31%)	*n* = 7 (35%)
Medium	*n* = 9 (22%)	*n* = 6 (19%)	*n* = 12 (33%)	*n* = 4 (20%)
Large	*n* = 4 (10%)	*n* = 3 (11%)	*n* = 3 (8%)	*n* = 2 (10%)
Extensive	*n* = 3 (7%)	*n* = 0 (0%)	*n* = 4 (11%)	*n* = 3 (15%)
*Any cartilage defect* ^a^				
Total	*n* = 51	*n* = 38	*n* = 55	*n* = 41
Fissure	*n* = 11 (22%)	*n* = 10 (26%)	*n* = 0 (0%)	*n* = 1 (2%)
Small	*n* = 13 (25%)	*n* = 10 (26%)	*n* = 11 (20%)	*n* = 13 (32%)
Medium	*n* = 11 (22%)	*n* = 9 (24%)	*n* = 13 (24%)	*n* = 10 (24%)
Large	*n* = 8 (16%)	*n* = 5 (13%)	*n* = 14 (26%)	*n* = 10 (24%)
Extensive	*n* = 8 (16%)	*n* = 4 (11%)	*n* = 17 (31%)	*n* = 7 (17%)

__^a^Partial thickness parts and full thickness parts.

**Table 4:** Cohen's kappa values for interobserver reliability.

Parameter	CTA	MRI
	Interobserver agreement ± SEM	Interobserver agreement ± SEM
*Full thickness cartilage defect*		
Presence	0.72 ± 0.05	0.55 ± 0.07
Size	0.47 ± 0.05	0.55 ± 0.04
*Any cartilage defect*		
Presence	0.82 ± 0.05	0.55 ± 0.06
Size	0.48 ± 0.05	0.50 ± 0.04
*Defect of the subchondral bone*		
Presence	0.70 ± 0.06	0.78 ± 0.04
Depth	0.60 ± 0.05	0.75 ± 0.04
Size	0.48 ± 0.04	0.63 ± 0.04
